# Temporal Variability in Electrocardiographic Indices in Subjects With Brugada Patterns

**DOI:** 10.3389/fphys.2020.00953

**Published:** 2020-09-03

**Authors:** Sharen Lee, Jiandong Zhou, Tong Liu, Konstantinos P. Letsas, Sandeep S. Hothi, Vassilios S. Vassiliou, Guoliang Li, Adrian Baranchuk, Raymond W. Sy, Dong Chang, Qingpeng Zhang, Gary Tse

**Affiliations:** ^1^Laboratory of Cardiovascular Physiology, Li Ka Shing Institute of Health Sciences, Hong Kong, China; ^2^School of Data Science, City University of Hong Kong, Hong Kong, China; ^3^Tianjin Key Laboratory of Ionic-Molecular Function of Cardiovascular Disease, Department of Cardiology, Tianjin Institute of Cardiology, Second Hospital of Tianjin Medical University, Tianjin, China; ^4^Second Department of Cardiology, Laboratory of Cardiac Electrophysiology, Evangelismos General Hospital of Athens, Athens, Greece; ^5^Heart and Lung Centre, New Cross Hospital, Wolverhampton, United Kingdom; ^6^Norwich Medical School, University of East Anglia, Faculty of Medicine, Imperial College London, London, United Kingdom; ^7^Arrhythmia Unit, Department of Cardiovascular Medicine, First Affiliated Hospital of Xi’an Jiaotong University, Xi’an, China; ^8^Division of Cardiology, Kingston General Hospital, Queen’s University, Kingston, ON, Canada; ^9^Sydney Medical School, University of Sydney, Sydney, NSW, Australia; ^10^Department of Cardiology, Royal Prince Alfred Hospital, University of Sydney, Sydney, NSW, Australia; ^11^Xiamen Cardiovascular Hospital, Xiamen University, Xiamen, China

**Keywords:** Brugada, temporal, variability, repolarization, ECG

## Abstract

**Introduction:**

Patients with Brugada electrocardiographic (ECG) patterns have differing levels of arrhythmic risk. We hypothesized that temporal variations in certain ECG markers may provide additional value for risk stratification. The present study evaluated the relationship between temporal variability of ECG markers and arrhythmic outcomes in patients with a Brugada pattern ECG. Comparisons were made between low-risk asymptomatic subjects versus high-risk symptomatic patients with a history of syncope, ventricular tachycardia (VT) or ventricular fibrillation (VF).

**Methods:**

A total of 81 patients presenting with Brugada patterns were recruited. Serial ECGs and electronic health records from January 2004 to April 2019 were analyzed. Temporal variability of QRS interval, J point-T_peak_ interval (JTp), T_peak_-T_end_ interval (Tp-e), and ST elevation (STe) in precordial leads V1-3, in addition to RR-interval from lead II, was assessed using standard deviation and difference between maximum and minimum values over the serial ECGs.

**Results:**

Patients presenting with type 1 Brugada ECG pattern initially had significantly higher variability in JTp from lead V2 (SD: 33.5 ± 13.8 vs. 25.2 ± 11.5 ms, *P* = 0.009; max-min: 98.6 ± 46.2 vs. 78.3 ± 47.6 ms, *P* = 0.047) and ST elevation in lead V1 (0.117 ± 0.122 vs. 0.053 ± 0.030 mV; *P* = 0.004). Significantly higher variability in Tp-e interval measured from lead V3 was observed in the VT/VF group compared to the syncope and asymptomatic groups (SD: 20.5 ± 8.5 vs. 16.6 ± 7.3 and 14.7 ± 9.8 ms; *P* = 0.044; max-min: 70.2 ± 28.9 vs. 56.3 ± 29.0 and 43.5 ± 28.5 ms; *P* = 0.011).

**Conclusion:**

Temporal variability in ECG indices may provide additional value for risk stratification in patients with Brugada pattern.

## Introduction

Brugada syndrome (BrS) is an ion channelopathy which results in characteristic electrocardiographic (ECG) changes and predisposes patients with increased risk of ventricular tachycardia (VT), ventricular fibrillation (VF), and sudden cardiac death (SCD). The stratification of SCD risk is influenced by many factors, primarily the ECG pattern and symptoms presented (Gourraud et al., 2017). Syncope, VT and VF are deemed as strong predictors of SCD, while asymptomatic patients are considered to be of relatively low risk (Sieira et al., 2016). ECG markers are being explored for early identification of high-risk patients before they become symptomatic (Asvestas et al., 2018).

The spatial dispersion of different ECG indices for risk stratification has been well-explored. However, little analysis has been performed on their temporal variability. A recent study has reported significant temporal variability in indices measured from serial ECGs, with greater variability in patients with type 1 pattern (Castro Hevia et al., 2019). In the past, temporal variability in ECG indices has been simply attributed to the recognized dynamicity in Brugada ECG pattern (BrP) (Bayes de Luna et al., 2012). The recent demonstration of the difference in temporal variability between patient subgroups (Gray et al., 2017) inspired our interest to examine the potential use of temporal variability concerning risk stratification. Hence, the present study aims to examine the difference in temporal variability of ECG markers for risk stratification between low-risk asymptomatic patients, and high-risk symptomatic patients with a history of syncope, VT, or VF.

## Methods

### Patient Selection and Electrocardiographic Measurement

This study received Ethics Approval from The Joint Chinese University of Hong Kong – New Territories East Cluster Clinical Research Ethics Committee (CREC Ref. No.: 2019.338). Institutional board approval was obtained and waived the need for patient consent in this retrospective study. The present single-center cohort consists of patients who visited the Prince of Wales Hospital, Hong Kong, China from the 1st of January, 2004 to the 1st of April, 2019 with electrocardiogram showing type 1 (cove-shaped) or type 2 (saddle-shaped) Brugada patterns. Patients with zero or one electronically documented ECG were excluded. Discrete ECGs throughout the follow-up duration were analyzed. The following ECG indices for leads V1, V2, and V3 from 12-lead ECG were measured using Philips ECGVue (standard edition): (1) QRS interval; (2) J point-T_peak_ interval (J-Tp, interval from J point to the peak of T wave); (3) T_peak_-T_end_ interval (Tp-e, interval from the peak to the end of T wave returning to baseline); and (4) ST elevation (STe). RR interval was measured from lead II. ECGs were excluded if (1) artefacts were present in leads V1-3; (2) QRS complex and T waves were unidentifiable; and (3) the rhythm was paced.

The patient history was reviewed to identify the presence of syncope, VT and VF. The cohort was divided into three groups based on symptoms documented during follow-up: (1) asymptomatic, (2) syncope, and (3) VT/VF. Patients who presented with both syncope and VT/VF were included in both groups. Patients who presented with type 1 Brugada ECG, and those who did not, were also compared. The average and temporal dispersion of individual ECG indices were calculated. Temporal dispersion was calculated by two methods: (1) maximum – minimum (max-min) value of the index; and (2) standard deviation (SD) of the index over the series of ECG taken.

### Statistical Analysis

Statistical analysis was performed using Stata MP 13. Statistical significance is defined as *P*-value <0.05. Intergroup differences were compared by Kruskal-Wallis’ two-way ANOVA and Fisher’s exact test for continuous and discrete variables, respectively. Pairwise comparison was performed for indices with significant intergroup differences by repeating the Kruskal-Wallis two-way ANOVA. Intra- and inter-observer agreement was assessed by the same investigator 4 weeks apart, and by a second investigator (Supplementary Material). by one-way mixed-effects individual, and two-way random-effects average absolute-agreement intraclass correlation coefficient (ICC), respectively (0 < ICC < 0.4 = poor; 0.4 < ICC < 0.59 = fair; 0.6 < ICC < 0.74 = good; 0.75 < ICC < 1.00 = excellent) (Cicchetti, 1994).

## Results

After excluding patients with fewer than two ECG records, the cohort comprised 81 patients (initially type 1 = 35.8%, median follow-up period = 1281 days, interquartile range of follow-up period = 2571 days, 1150 ECGs analyzed, female = 8.6%, age of first BrP = 52.5 ± 1.85, age interquartile range = 24.0 years). All patients were Han Chinese and 79 of the 81 subjects were probands. The remaining two patients were identified from family screening. The age of first BrP and patient sex of the subgroups did not differ significantly (Supplementary Table 1). Both intra- and inter-observer agreement achieved fair to excellent quality (ICC > 0.4; Supplementary Tables 2, 3).

Electrocardiographic indices were compared between patients with an initial type 1 pattern and those who had non-type 1 patterns (Table 1). The initial type 1 group displayed significantly longer lead V3 QRS interval (*P* = 0.021), shorter lead V3 JTp (*P* = 0.011), and greater lead V1 ST elevation (*P* = 0.000) compared to the non-type 1 group. They also showed significantly greater temporal dispersion for both lead V2 JTp (*P*-value: SD = 0.009, max-min = 0.047) and lead V1 ST elevation (*P*-value: SD = 0.004, max-min = 0.001).

**TABLE 1:** ECG biomarkers for initial type 1 vs. non-type 1 patterns.

	**Overall (*n* = 81)**	**Non-type 1 (*n* = 52)**	**Type 1 (*n* = 29)**	***P*-value**

**Average**				
RR	863112	880118	83395.2	0.096
QRSV1	10413.5	10213.4	10713.3	0.085
QRSV2	11015.0	10815.8	11313.1	0.127
QRSV3	11114.8	10815.6	11512.4	**0.021**
JTpV1	20226.7	20329.4	20121.2	0.436
JTpV2	18125.9	18126.2	18025.9	0.825
JTpV3	17523.9	18024.7	16619.9	**0.011**
TpeV1	83.715.4	82.614.6	85.616.7	0.640
TpeV2	10114.5	10214.5	10114.8	0.598
TpeV3	10514.6	10613.3	10316.8	0.220
STeV1	0.1460.143	0.1010.081	0.2270.189	**0.000**
STeV2	0.2970.159	0.2770.139	0.3340.187	0.285
STeV3	0.1380.080	0.1330.071	0.1490.096	0.910

**Dispersion: SD**				

RR	86.640.4	84.843.3	89.935.3	0.509
QRSV1	12.18.03	11.06.12	14.110.5	0.241
QRSV2	14.69.73	14.210.1	15.29.24	0.503
QRSV3	10.94.73	10.44.80	11.94.55	0.138
JTpV1	23.713.3	25.314.1	20.911.4	0.215
JTpV2	28.212.9	25.211.5	33.513.8	**0.009**
JTpV3	21.413.1	20.710.9	22.616.5	0.922
TpeV1	17.37.59	18.18.25	15.96.11	0.428
TpeV2	20.610.2	19.29.31	23.211.4	0.126
TpeV3	16.08.83	15.26.80	17.411.6	0.828
STeV1	0.0760.082	0.0530.030	0.1170.122	**0.004**
STeV2	0.1270.087	0.1230.089	0.1350.084	0.419
STeV3	0.0700.050	0.0650.052	0.0790.046	0.159

**Dispersion: Max-Min**				

RR	277155	274163	280144	0.863
QRSV1	39.728.5	35.725.6	46.832.2	0.153
QRSV2	46.631.5	43.028.5	53.035.8	0.268
QRSV3	34.820.4	33.120.5	37.820.3	0.339
JTpV1	75.545.3	78.846.7	69.442.8	0.342
JTpV2	85.647.9	78.347.6	98.646.2	**0.047**
JTpV3	66.144.6	63.638.9	70.553.8	0.957
TpeV1	53.827.1	55.629.2	50.623.0	0.509
TpeV2	63.535.5	58.233.1	73.038.2	0.081
TpeV3	50.829.2	48.427.3	55.032.4	0.454
STeV1	0.2100.225	0.1460.123	0.3240.310	**0.001**
STeV2	0.3670.267	0.3380.251	0.4170.292	0.204
STeV3	0.1910.145	0.1750.138	0.2210.154	0.239

*Units in milliseconds or millivolts. Variables with *P* < 0.05 are shown in bold text.*

Subsequent analyses were conducted by comparing ECG indices between asymptomatic patients (*n* = 39), patients with syncope (*n* = 38), and patients with VT/VF (*n* = 13) group. Within the syncope and VT/VF subgroups, five and two patients in the respective groups were initially asymptomatic, and developed syncope or VT/VF over the course of follow-up. A significant increase in variability was found in Tp-e measured form lead V3 under both standard deviation (*P* = 0.044) and maximum – minimum (*P* = 0.011) calculations, as shown in Table 2. Under further pairwise comparison, VT/VF group had significantly greater temporal dispersion in comparison to the asymptomatic group with both standard deviation (*P* = 0.021) and maximum – minimum (*P* = 0.007) methods, and to the syncope group under maximum- minimum (*P* = 0.046) calculation. Furthermore, the average extent of STe in lead V3 is significantly higher in VT/VF group in comparison to both the syncope (*P* = 0.039) and the asymptomatic group (*P* = 0.010) under pairwise comparison.

**TABLE 2:** ECG biomarkers categorized based on symptoms.

	**Asymptomatic (*n* = 39)**	**Syncope (*n* = 38)**	**VT/VF (*n* = 13)**	***P*-value**

**Average**				
RR	870104	857126	835103	0.564
QRSV1	10513.8	10313.8	10617.5	0.712
QRSV2	10915.4	11115.5	11517.1	0.631
QRSV3	11115.6	11114.9	11416.1	0.873
JTpV1	20030.2	20523.3	20327.2	0.841
JTpV2	19127.6	18225.2	17731.8	0.706
JTpV3	17323.0	17924.8	16831.9	0.185
TpeV1	86.717.5	80.412.7	86.715.1	0.203
TpeV2	10516.6	97.812.1	1009.73	0.121
TpeV3	10815.2	10214.2	10810.3	0.193
STeV1	0.1350.132	0.1640.159	0.1680.159	0.922
STeV2	0.3260.168	0.2690.145	0.2540.153	0.111
STeV3	0.1600.090	0.1200.066	0.0970.051	**0.017**

**Dispersion: SD**				

RR	85.046.0	88.036.1	74.429.8	0.417
QRSV1	13.29.9	11.25.4	13.96.37	0.487
QRSV2	13.59.9	16.39.8	12.46.6	0.259
QRSV3	10.95.0	11.44.6	9.583.6	0.397
JTpV1	23.713.5	24.713.6	21.47.6	0.876
JTpV2	30.515.5	25.99.6	26.69.7	0.598
JTpV3	23.316.3	20.19.4	19.410.1	0.731
TpeV1	17.78.90	17.26.5	16.55.8	0.929
TpeV2	20.912.0	20.38.5	21.46.6	0.725
TpeV3	14.79.8	16.67.3	20.58.5	**0.044**
STeV1	0.0730.065	0.0820.100	0.0650.041	0.984
STeV2	0.1340.090	0.1220.086	0.1180.074	0.778
STeV3	0.0810.061	0.0610.037	0.0640.039	0.201

**Dispersion: Max-Min**				

RR	251163	300153	288118	0.125
QRSV1	40.632.3	40.025.1	49.126.2	0.467
QRSV2	39.529.9	55.632.5	47.927.4	0.077
QRSV3	31.820.2	38.921.0	37.120.8	0.270
JTpV1	69.843.1	83.748.4	77.534.1	0.460
JTpV2	83.551.1	87.746.5	95.847.2	0.638
JTpV3	66.052.8	67.337.2	71.845.6	0.565
TpeV1	50.627.8	57.727.6	58.423.0	0.535
TpeV2	57.233.4	69.637.6	80.937.9	0.080
TpeV3	43.528.5	56.329.0	70.228.9	**0.011**
STeV1	0.1820.150	0.2500.287	0.2380.218	0.835
STeV2	0.3490.236	0.2890.306	0.4000.277	0.894
STeV3	0.2150.160	0.1740.133	0.2080.171	0.405

*Units in milliseconds or millivolts. Variables with *P* < 0.05 are shown in bold text.*

## Discussion

The present study demonstrates that increased temporal variability in Tp-e is found in patients with Brugada pattern of higher SCD risk. Tp-e has been used to quantify the transmural dispersion of ventricular repolarization and used in BrS risk-stratification (Xia et al., 2005; Tse et al., 2018a). Increased transmural dispersion prolongs ventricular repolarization, which can lead to re-entry in phase 2 and subsequently ventricular tachyarrhythmia (Yan and Antzelevitch, 1999). Although the dynamicity of the Brugada pattern is well known, the significance of temporal variability in the risk stratification of BrS patients has not been fully explored (Viskin et al., 2018). The significant temporal variability in Tp-e amongst the high-risk arrhythmic group suggests the presence of a temporal variation in the transmural repolarization dispersion, and increased ventricular repolarization instability may explain the marked increase SCD risk in the symptomatic group. The fluctuations in the extent of transmural dispersion are further supported by the insignificant difference in Tp-e, and the dynamic manifestation of Brugada ECG pattern (Antzelevitch et al., 2005). Differing with the study by Castro Hevia et al. (2019), significant temporal variability was not noted in QRS duration in the present study, which might be attributed to the difference in calculation methodology. The significant intergroup differences in the average value of ECG indices is due to the diverse electrocardiographic pattern.

Although the diagnosis of Brugada syndrome focuses on the characteristic ECG pattern in leads V1 and V2, the present study demonstrates that lead V3 can also be useful in risk stratification. Information from lead V3 is often considered to be redundant in the clinical diagnosis of Brugada syndrome since it is less sensitive than leads V1-2 for Brugada ECG changes (Govindan et al., 2010). However, the present findings suggest that lead V3 might be able to provide insights into risk stratification through its sensitivity for ECG variability, and for which further research is needed to explore its clinical value in variability detection. It has been reported that Tp-e is longest in V3 when measured in children, possibly due to a higher number of M cells distributed in the interventricular septum (Bieganowska et al., 2013). The difference in temporal variability across the leads highlights the heterogeneity in ventricular repolarization of Brugada patients. Further research on the spatiotemporal dispersion of ECG indices is needed to elucidate the dynamicity in Brugada pattern.

The insignificant difference between the asymptomatic and syncope groups could be explained by the exact etiology of syncope. It has been reported that whilst patients presenting with unexplained syncope and a positive electrophysiological study are at high risk for arrhythmia, those with neurally mediated syncope share a similar prognosis to asymptomatic patients (Giustetto et al., 2017). Furthermore, there is growing evidence for similar genetic mutations to be present in both epilepsy and BrS. Syncope of such potential non-cardiogenic origins is unlikely to be affected by temporal variability of cardiac conduction and repolarisation, which might explain the insignificant results (Parisi et al., 2013; Tiron et al., 2015).

To reduce bias in ECG analysis, automatic measurements by the ECG machine can be applied. However, a comparison should be made against manual measurements to confirm accuracy in the detection of different ECG features, for example, the J point in Brugada ECGs. Future studies should further evaluate the use of automatic measurements in all ECG parameters analyzed in the present study and assess the clinical applications of the ECG parameters, particularly Tp-e. Whilst clinical studies have demonstrated that prolonged Tp-e reflects the presence of a resolved, temporary, arrhythmogenic substrate in patients with acute myocardial infarction, and predicts mortality for these patients, further research is required to explore its applicability in outcome prediction for spontaneous arrhythmia due to a channelopathy (Haarmark et al., 2009; Elitok et al., 2015).

### Strengths and Limitations

There are several notable strengths of the present study. Firstly, a comprehensive analysis of different ECG features has been performed, with 3–50 ECGs measured per patient, and a total of more than 1000 ECGs analyzed. This extended previous studies using only single ECG measurements at baseline (Tse et al., 2018b; Lee et al., 2020). Moreover, there is good inter and intra-observer variability, as reflected by the intra-class coefficient. Several limitations should also be noted for the present study. First, the cohort is small and from a single center, hence findings should be externally validated and tested in a separate larger cohort in the future. Gender differences were not explored in the present study due to the limited number of female patients (*n* = 7). Since the majority of the patients were distributed in the middle ages (median age of first Brugada BrP onset = 53.0 years, interquartile range = 20.0 years), in addition to the relatively small cohort, the age differences were not examined. Unfortunately, only two patients undergone genetic testing in the present cohort, hence their genetic status cannot be commented upon. Moreover, the retrospective nature of the present study is susceptible to the bias inherent to such analyses. Besides, the sensitivity of the caliper used in measurement is limited by its one-decimal-place display, which results in a larger error in measuring small values, such as STe. The intervals between serial ECGs taken is variable between patients, and within a single patient, with more ECGs taken when the patients are less stable. Therefore, the ECG indices measured may tend to reflect a state of instability Errors may also be introduced in view of different operators undertaking the ECGs on different days. Furthermore, the relatively short follow-up duration limits the predictive value of the ECG parameters found toward patient life expectancy.

## Conclusion

The present study demonstrates the presence of increased temporal variability in Tp-e amongst high-risk BrS patients, which illustrates a potential role for the analysis of serial changes in the surface ECG in the prognostic workup of BrS. Further research into the spatiotemporal dispersion of Brugada ECG patterns is need to gain insights into the dynamicity of Brugada electrophysiological changes.

## Data Availability Statement

The anonymized dataset and relevant materials have been made publicly available at Zenodo and can be accessed here: https://zenodo.org/record/3351892; https://zenodo.org/record/3266172; and https://zenodo.org/record/3266179.

## Ethics Statement

The studies involving human participants were reviewed and approved by The Joint Chinese University of Hong Kong – New Territories East Cluster Clinical Research Ethics Committee (The Joint CUHK-NTEC CREC). Written informed consent for participation was not required for this study in accordance with the national legislation and the institutional requirements.

## Author Contributions

SL and GT contributed to the study conception, data acquisition, database building, statistical analysis, manuscript drafting, and manuscript revision. All authors contributed to the data interpretation, statistical analysis, and manuscript revision.

## Conflict of Interest

The authors declare that the research was conducted in the absence of any commercial or financial relationships that could be construed as a potential conflict of interest.
