# Supplementary material for: Temporal Variability in Electrocardiographic Indices in Subjects With Brugada Patterns
Source: Front Physiol. 2020 Sep 3;11:953. doi: 10.3389/fphys.2020.00953 (PMC7494959; doi:10.3389/fphys.2020.00953)
Supplement: Supplementary file 1 [file Data_Sheet_1.docx]

Supplementary Table 1. Age of First BrP and Sex of the Study Cohort

|  | Overall (n=81) | Non-Type 1 (n=52) | Type 1 (n-29) | P-Value |
| --- | --- | --- | --- | --- |
| Female | 8.64% (n=7) | 11.5% (n=6) | 3.45% (n=1) | 0.412 |
| Age of First BrP | 52.5 ± 1.85 | 51.6 ± 2.60 | 53.9 ± 2.38 | 0.631 |
|  | | | | |
|  | Asymptomatic | Syncope | VT/VF | P=-Value |
| Female | 17.9% (n=7) | 7.89% (n=3) | 7.69% (n=1) | 1.00 |
| Age of First BrP | 51.7 ± 2.59 | 52.4 ± 2.74 | 50.4 ± 5.53 | 0.902 |

Supplementary Table 2. Intra-observer Intraclass Correlation Coefficient

| Parameter | Individual Intraclass Correlation Coefficient (95% Confidence Interval) |
| --- | --- |
| RR | 0.996 [0.993, 0.998] |
| QRS V1 | 0.905 [0.840, 0.945] |
| QRS V2 | 0.634 [0.438, 0.773] |
| QRS V3 | 0.737 [0.581, 0.840] |
| JTp V1 | 0.906 [0.842, 0.945] |
| JTp V2 | 0.504 [0.270, 0.683] |
| JTp V3 | 0.611 [0.408, 0.758] |
| Tp-e V1 | 0.557 [0.336, 0.720] |
| Tp-e V2 | 0.681 [0.502, 0.804] |
| Tp-e V3 | 0.573 [0.356, 0.731] |
| STe V1 | 0.857 [0.763, 0.916] |
| STe V2 | 0.773 [0.634, 0.863] |
| STe V3 | 0.744 [0.592, 0.845] |

Supplementary Table 3. Inter-observer Intraclass Correlation Coefficient

| Parameter | Average Intraclass Correlation Coefficient (95% CI) |
| --- | --- |
| RR | 0.872 [0.763, 0.931] |
| QRS V1 | 0.438 [-0.045, 0.698] |
| QRS V2 | 0.457 [-0.011, 0.708] |
| QRS V3 | 0.647 [0.343, 0.810] |
| JTp V1 | 0.409 [-0.100, 0.682] |
| JTp V2 | 0.770 [0.572. 0.876] |
| JTp V3 | 0.574 [0.208, 0.771] |
| Tp-e V1 | 0.769 [0.569, 0.876] |
| Tp-e V2 | 0.762 [0.613, 0.888] |
| Tp-e V3 | 0.732 [0.501, 0.856] |
| STe V1 | 0.835 [0.693, 0.911] |
| STe V2 | 0.943 [0.895, 0.970] |
| STe V3 | 0.995 [0.991, 0.997] |
