# Supplementary material for: Temporal Variability in Electrocardiographic Indices in Subjects With Brugada Patterns
Source: Front Physiol. 2020 Sep 3;11:953. doi: 10.3389/fphys.2020.00953 (PMC7494959; doi:10.3389/fphys.2020.00953)
Supplement: Supplementary file 2 [file data_sheet_2.docx]

**Supplementary Figures**


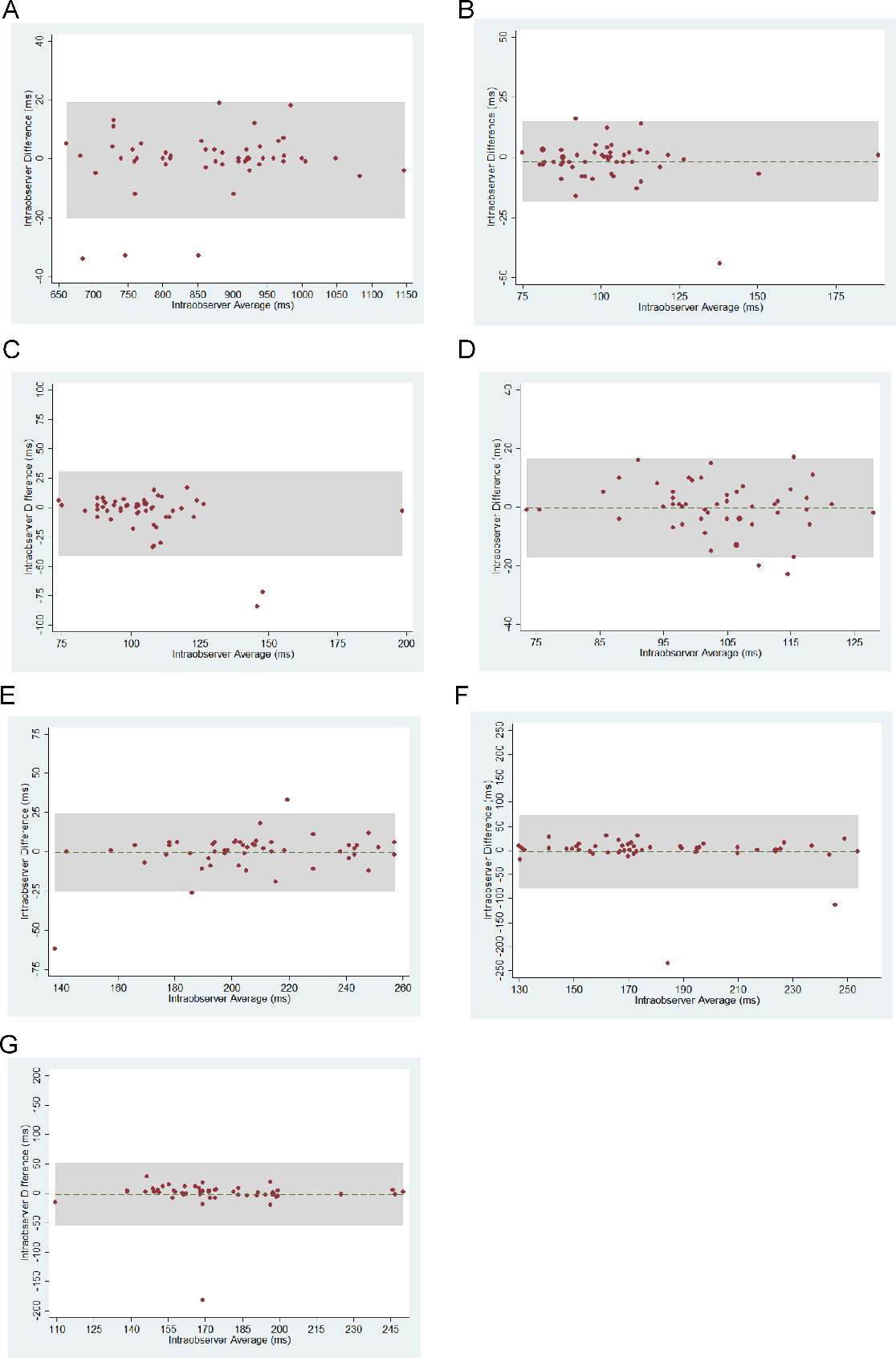


Supplementary Figure 1. Intra-observer variability for RR interval (A), QRS measured from V1 (B), V2 (C) and V3 (D), JTp measured from V1 (E), V2 (F) and V3 (G).


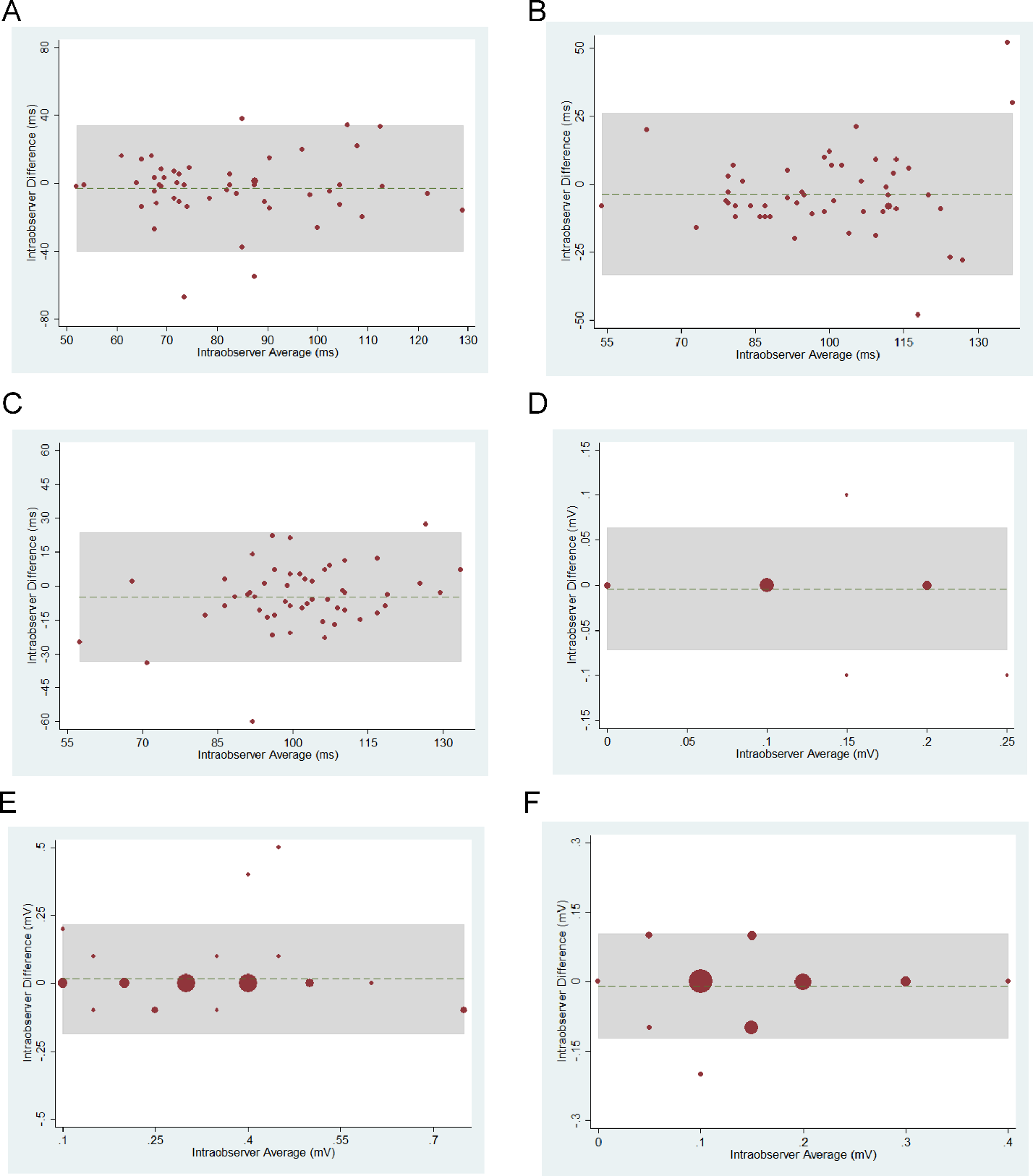


Supplementary Figure 2. Intra-observer variability for Tp-e from V1 (A), V2 (B) and V3 (C), STe measured from V1 (D), V2 (E) and V3 (F).


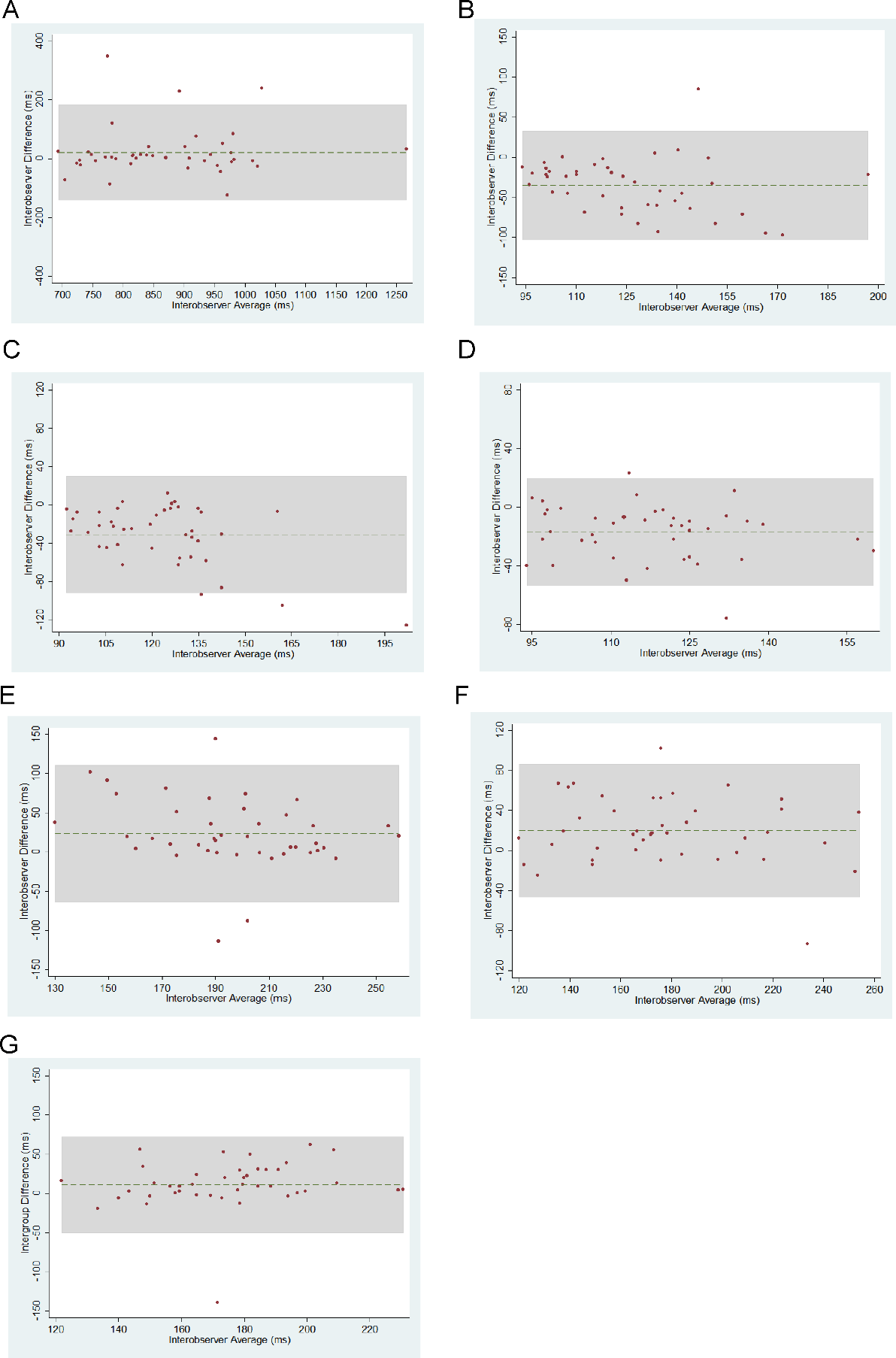


Supplementary Figure 3. Inter-observer variability for RR interval (A), QRS measured from V1 (B), V2 (C) and V3 (D), JTp measured from V1 (E), V2 (F) and V3 (G).


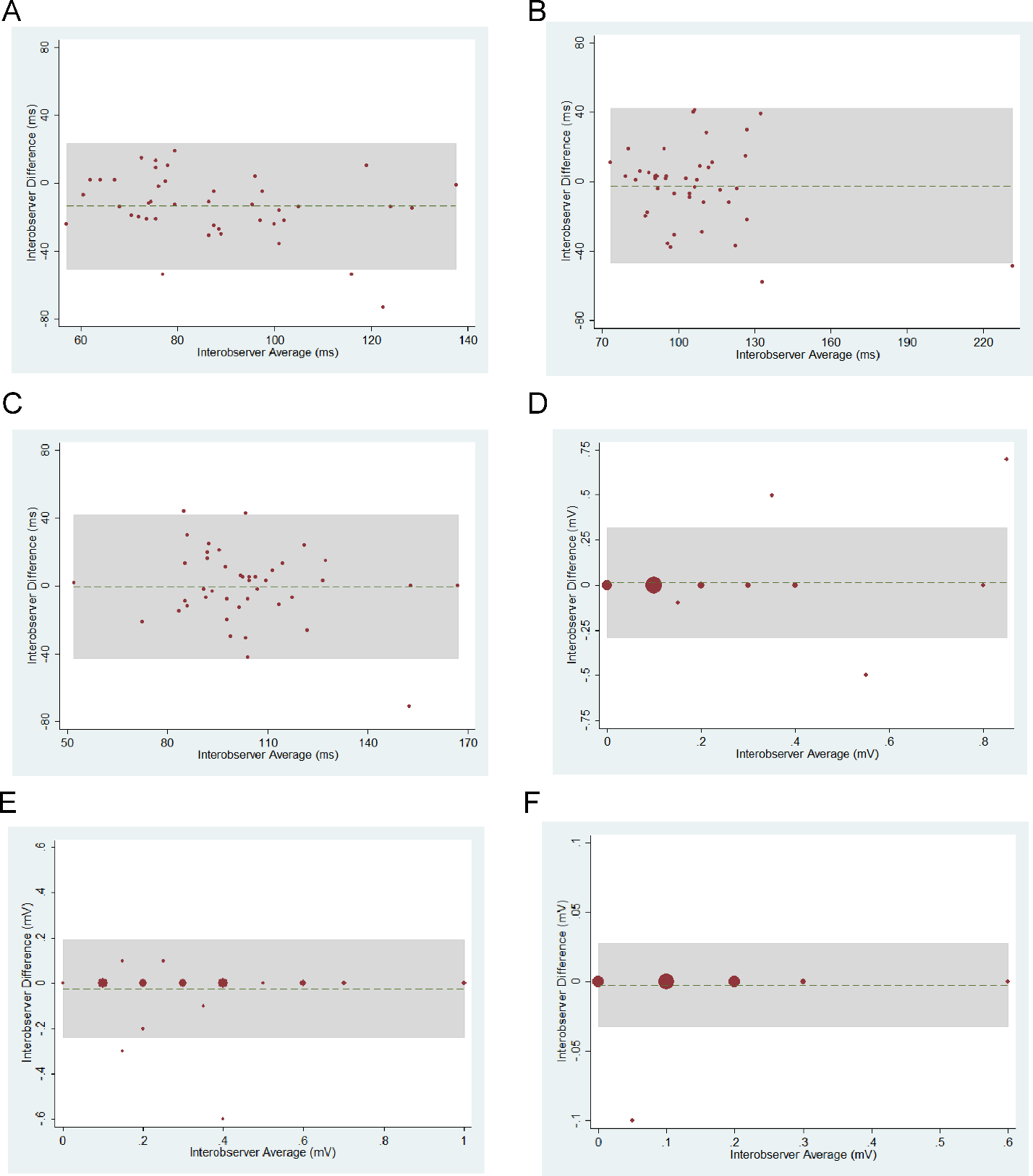


Supplementary Figure 4. Inter-observer variability for Tp-e from V1 (A), V2 (B) and V3 (C), STe measured from V1 (D), V2 (E) and V3 (F).
